# NETosis in Alzheimer’s Disease

**DOI:** 10.3389/fimmu.2017.00211

**Published:** 2017-03-02

**Authors:** Enrica Caterina Pietronigro, Vittorina Della Bianca, Elena Zenaro, Gabriela Constantin

**Affiliations:** ^1^Department of Medicine, Section of General Pathology, University of Verona, Verona, Italy

**Keywords:** Alzheimer’s disease, neutrophils, neutrophil extracellular traps, blood-brain barrier, neuroinflammation

## Abstract

Alzheimer’s disease (AD) is a neurodegenerative disorder characterized by the progressive deterioration of cognitive functions. Its neuropathological features include amyloid-β (Aβ) accumulation, the formation of neurofibrillary tangles, and the loss of neurons and synapses. Neuroinflammation is a well-established feature of AD pathogenesis, and a better understanding of its mechanisms could facilitate the development of new therapeutic approaches. Recent studies in transgenic mouse models of AD have shown that neutrophils adhere to blood vessels and migrate inside the parenchyma. Moreover, studies in human AD subjects have also shown that neutrophils adhere and spread inside brain vessels and invade the parenchyma, suggesting these cells play a role in AD pathogenesis. Indeed, neutrophil depletion and the therapeutic inhibition of neutrophil trafficking, achieved by blocking LFA-1 integrin in AD mouse models, significantly reduced memory loss and the neuropathological features of AD. We observed that neutrophils release neutrophil extracellular traps (NETs) inside blood vessels and in the parenchyma of AD mice, potentially harming the blood–brain barrier and neural cells. Furthermore, confocal microscopy confirmed the presence of NETs inside the cortical vessels and parenchyma of subjects with AD, providing more evidence that neutrophils and NETs play a role in AD-related tissue destruction. The discovery of NETs inside the AD brain suggests that these formations may exacerbate neuro-inflammatory processes, promoting vascular and parenchymal damage during AD. The inhibition of NET formation has achieved therapeutic benefits in several models of chronic inflammatory diseases, including autoimmune diseases affecting the brain. Therefore, the targeting of NETs may delay AD pathogenesis and offer a novel approach for the treatment of this increasingly prevalent disease.

## Introduction

The formation of neutrophil extracellular traps (NETs) is a defense mechanism used by neutrophils to trap and efficiently limit the damage caused by a wide range of microbial targets (1). NET production is associated with dramatic changes in cellular morphology, including the extrusion of decondensed chromatin into the extracellular space to form web-like structures decorated with histones and granular antimicrobial proteins, such as neutrophil elastase (NE), myeloperoxidase (MPO), proteinase 3, cathepsin G, lactoferrin, matrix metalloproteinase 9 (MMP-9), peptidoglycan-recognition proteins, pentraxin, and LL-37 (1–4). The sequential molecular events that generate NETs are the production of reactive oxygen species (ROS), the migration of NE protease and later MPO from granules to the nucleus, the processing of histones, and the rupture of the cell (5). NETs provide a key defense mechanism against pathogens to prevent their systemic dissemination during infection (1, 6). However, NETs are also major effectors involved during sterile inflammation, autoimmune diseases, such as systemic lupus erythematosus (SLE), and atherosclerosis, and they may also promote metastasis (1, 7). In addition, we have recently shown the release of NETs in Alzheimer’s disease (AD), suggesting that NETs may also play a role in AD pathology (8).

Alzheimer’s disease, one of the most devastating neurodegenerative disorders, is characterized by progressive memory decline and cognitive deficits. The main neuropathological features of AD include neuritic plaques formed by deposits of amyloid-β (Aβ), the abnormal accumulation of hyper-phosphorylated tau protein in the neuronal soma, which manifests as neurofibrillary tangles (NFTs), synaptic dysfunction, and neuronal loss (9). AD is also characterized by cerebral amyloid angiopathy due to Aβ deposits in the cerebral vasculature, which lead to luminal stenosis, endothelial damage, basement membrane thickening, thrombosis, loss of autoregulation, and vasospasm (10). Chronic neuroinflammation is thought to play a role in AD pathology, and recent studies have identified several inflammation pathway genes associated with the risk of AD (11, 12). Microglial activation precedes neuronal loss in AD patients, and microglia-mediated neuro-inflammatory responses may promote the neurodegeneration observed in AD (9, 13). Moreover, in response to Aβ or NFTs, microglial cells produce pro-inflammatory cytokines, chemokines, and complement peptides, which can amplify the neuroinflammation in AD (13). Aβ and tau deposits cause detrimental effects in the neuronal milieu, due to the excessive release of cytotoxic factors, including the interleukins IL-1β and IL-6, tumor necrosis factor α (TNFα), and free radicals, enhancing neuroinflammation and neuronal damage (8, 11–14). Epidemiological studies indicate that non-steroidal anti-inflammatory drugs (NSAIDs) reduce the risk of AD, providing further evidence that inflammation mechanisms play a role in this disease (15, 16). However, the lack of efficacy of NSAIDs against AD in clinical trials suggests that more specific inflammation mechanisms must be identified to inhibit AD-related neuroinflammation (11, 16, 17).

Compelling evidence indicates that AD-related inflammation develops in two different but interconnected compartments: the blood and the brain. In this context, systemic inflammation can lead to “brain activation,” whereas cerebral inflammation may in turn influence the peripheral system through the release of danger signals and other inflammatory mediators (12, 18–21). The blood–brain barrier (BBB) is a connection point between blood and circulating leukocytes on one side and the brain parenchyma on the other. It is a highly specialized endothelial cell membrane that regulates the passage of essential nutrients and leukocytes into the central nervous system (CNS) and facilitates the clearance of potentially neurotoxic molecules from the brain to the blood (18–21). The BBB together with vascular cells (pericytes and vascular smooth muscle cells), glial cells, and neurons, constitutes the neurovascular unit (NVU) (20, 21). AD is characterized by the loss of BBB integrity, which disrupts the clearance of Aβ and thus promotes Aβ accumulation in the brain, leading to neuronal injury and cognitive decline (20). The accumulation of Aβ in the brain and in the vessel walls also induces the expression of adhesion molecules on brain endothelial cells and the release of inflammatory mediators, such as cytokines, chemokines, and complement system peptides, potentially facilitating the adhesion and subsequent transmigration of leukocytes. Previous studies have shown that tau may also contribute to BBB deterioration *in vitro*, and BBB dysfunction correlates with the appearance of perivascular tau around major hippocampal blood vessels *in vivo* (22–24). Both tau and Aβ may, therefore, induce BBB dysfunction, contributing to brain inflammation and neurodegeneration.

The role of circulating immune system cells in AD has not been investigated in detail, but the migration of cells related to both innate and adaptive immunity has been observed in the AD brain (21, 25, 26). For example, monocytes migrate through the brain endothelium into the AD brain in a CCR2-dependent manner, and previous studies suggest they may promote Aβ clearance (27). However, the replacement of brain-resident myeloid cells with circulating peripheral monocytes in AD mouse models showed that monocyte repopulation does not modify the amyloid load, challenging the idea that peripheral monocytes play a role in Aβ clearance (28, 29). Lymphocytes can also enter the AD brain, and both CD4^+^ and CD8^+^ T cells in AD patients were shown to adhere inside the cerebral blood vessels or to migrate into the parenchyma (21, 26). Nevertheless, the role of these cells remains unclear because recent studies indicate they may play either a positive or negative role in AD models, probably depending on the specific cell subset and disease phase (21, 26). Unexpectedly, neutrophils were also found inside the brain vessels and parenchyma of AD subjects, and the capacity of neutrophils to invade the AD brain has recently been confirmed (8, 30–32). Moreover, our recent data reveal that neutrophils adhere to blood vessels and infiltrate inside the brain parenchyma in two transgenic animal models of AD, inducing cognitive deficit and neuropathological changes (8). Neutrophil depletion reduced the neuropathological hallmarks of AD and improved memory functions in these models, suggesting that neutrophils play a key role in AD pathogenesis (8).

Recently, we have observed NETs within the cerebral vasculature and parenchyma of animal AD models and individuals with AD, suggesting that NETs can potentially harm the BBB and neural cells (8). In this review, we discuss the involvement of NETs in AD as a novel mechanism for neutrophil-mediated neurotoxicity and neurodegeneration and suggest that the inhibition of NETs may offer a new pharmacological approach to slow down the progression of this disease.

## Neutrophils in AD

Circulating neutrophils are the most abundant leukocytes in the peripheral blood and they provide the first line of defense in the innate immune system. Neutrophils are short-lived cells with circulating half-lives of approximately 1.5 h in mice and 8 h in humans, although this was recently challenged and longer survival times of up to several days were reported in humans (1). Nevertheless, neutrophils are activated during inflammation and their longevity increases, allowing them to carry out more complex activities, potentially causing bystander cell injury. Neutrophils are thought to be the main protagonists in the first line of defense during acute inflammation, when many of these cells migrate into tissues and can easily be identified using conventional histology techniques. However, neutrophils have attracted more attention recently in the context of chronic inflammation, e.g., atherosclerosis, rheumatoid arthritis, SLE, anti-neutrophil cytoplasmic antibody-related vasculitis, deep vein thrombosis, chronic obstructive pulmonary disease, cystic fibrosis, and animal models of multiple sclerosis (33–35). Neutrophils are now thought to be key players that directly affect the pathogenesis of chronic inflammatory diseases. For example, they were recently shown to play a prominent role in chronic low-grade adipose tissue inflammation and insulin resistance mediated by the secretion of elastase (36, 37).

Neutrophil recruitment in the CNS is a central process during the pathogenesis of several neuro-inflammatory disorders, ranging from bacterial and viral encephalitis to non-infectious conditions, such as cerebral ischemia, trauma, and demyelinating syndromes (25, 35, 38, 39). Previous studies have shown that neutrophils transmigrated in the CNS acquire a toxic phenotype and approach neuronal cells, where they release harmful molecules and can compromise neuronal functions (40). Therefore, limiting neutrophil migration and/or functions can positively influence the outcome of neuronal injuries (8, 21, 35, 39).

The first evidence that neutrophils accumulate in the CNS of AD patients was the detection of cells expressing the neutrophil-specific protease cathepsin G within the AD brain parenchyma and cerebral blood vessels, often associated with Aβ deposits (30). This was followed by the detection of CAP37, an inflammatory mediator constitutively expressed in neutrophils, in the cerebral microvasculature of AD patients but not in age-matched controls or patients with other neuropathological conditions, such as Pick’s disease, Parkinson’s disease, Binswanger’s disease, or supranuclear palsy (31, 41). Initially, the expression of CAP37 in the AD brain was not linked with the presence of neutrophils, but was instead associated with endothelial activation and neuronal cells, and was thought to be induced by Aβ (31, 41, 42). More recently, we identified MPO^+^ cells in areas with Aβ deposits, further supporting the presence of neutrophils in the AD brain (8). We found that intraparenchymal MPO^+^ cells were mainly localized to within 50 μm of Aβ plaques, and their distribution was non-random, suggesting that Aβ may act as a chemoattractant by creating a favorable microenvironment for the accumulation of neutrophils inside the brain, thus promoting their pro-inflammatory activities (8). We also used (i) hematoxylin and eosin staining to confirm the presence of polysegmented nuclei in cells that have migrated perivascularly or within the parenchyma (8), (ii) napthol AS-D chloroacetate esterase staining in brain sections to confirm the presence of cells of the granulocytic lineage specifically in AD brains (43, 44), and (iii) staining for the neutrophil-specific marker, CD66b, which likewise confirmed that neutrophils were present specifically in the brains of AD subjects but not age-matched controls (8). These neuropathological studies are supported by recent clinical data revealing increased numbers of neutrophils or a higher neutrophil/lymphocyte ratio associated with AD, suggesting that changes in the neutrophil population could be used as markers of AD-related peripheral inflammation (45–47). The amyloid protein precursor (APP) is also expressed more strongly in the granulocytes of AD patients compared to controls, whereas there was no statistically significant difference in the lymphocyte and mononuclear cell populations, suggesting that the strong expression of APP in peripheral mononuclear cells could be used for the early diagnosis of AD (48). Other studies have also revealed differences in neutrophil functions and changes in granulocyte density in AD patients, further suggesting that the analysis of blood neutrophils may offer new AD biomarkers (49, 50).

In agreement with the data from AD subjects, we have recently shown the presence of Gr-1^+^ cells in 3xTg-AD mice during the early phases of AD, and more recent data obtained in the 5xFAD transgenic AD model revealed Gr1^+^ cells infiltrating the brain parenchyma and migrating toward Aβ plaques (32, 51). Our data in 5xFAD and 3xTg-AD mice confirmed these results, showing infiltrating neutrophils within the brain parenchyma at the onset of memory deficit, especially in the cortex and hippocampus, highlighting the role of these cells in AD pathogenesis (8). In addition, our two-photon laser-scanning microscopy (TPLSM) studies revealed neutrophil extravasation inside the cerebral parenchyma during the early phases of AD, preferentially in zones adjacent to vascular and intraparenchymal Aβ deposits, suggesting that Aβ may play an important role in neutrophil recruitment and movement inside the brain parenchyma (8). As stated above, Aβ may act as a chemoattractant for neutrophils and may represent an FPR1-binding “end-target” chemoattractant prevailing over “intermediate” chemokines, potentially contributing to the directional bias observed for a significant proportion of extravasated neutrophils in the brains of AD mouse models (8, 52, 53). FPR1 and LFA-1 may, therefore, promote neutrophil deep tissue penetration and thus contribute to widespread tissue damage.

In our studies, LFA-1 integrin controlled not only the intraparenchymal motility of extravasated neutrophils but also their intravascular adhesion in the cerebral microcirculation of transgenic AD mice (8). By blocking LFA-1 integrin in AD mice during the early phases of AD, neutrophil adhesion and extravasation were prevented and the neuropathological hallmarks of AD were clearly reduced, thus restoring cognitive functions. Notably, a transient therapeutic blockade of LFA-1 integrin during the early stages of disease also provided a long-term beneficial effect on cognition in older mice, suggesting that the therapeutic reduction of neutrophil trafficking during the early phases of AD may have prolonged beneficial effects in AD patients. Moreover, 3xTg-AD mice lacking LFA-1 integrin showed improved memory functions in behavioral tests compared to wild-type control mice, and the severity of microgliosis was reduced (8). The role of neutrophils in AD has been defined only recently, so the mechanisms controlling neutrophil trafficking and interactions with CNS-resident cells must be investigated in more detail.

*In vivo* experiments have shown that fluorescence-labeled neutrophils start to infiltrate the brain parenchyma of 5xFAD mice 12–18 h after cell injection, continue to migrate and reach a peak at 24 h post-injection, and then become undetectable at 48 h post-injection, suggesting that the half-life of the migrating neutrophils in the brain is approximately 12 h. Neutrophils are highly reactive cells, releasing ROS, enzymes, NETs, and cytokines, and can thus cause chronic collateral tissue damage even in the absence of substantial accumulation within tissues during low-grade chronic sterile inflammation. Furthermore, previous results from our group and others have shown that neutrophils do not necessarily need to accumulate in high numbers in order to induce tissue damage: intravascular adhesion *per se* without transmigration is sufficient to induce endothelial injury and the resulting tissue damage (54, 55). This is supported by our data showing that blocking LFA-1 integrin, which controls the intravascular adhesion of neutrophils, reduces cognitive damage and neuropathological lesions in AD models. Our recent results also demonstrate that transgenic AD mouse models treated with a neutrophil-depleting antibody show a reduction in both microglial cell density and their activation state, suggesting that neutrophils promote the activation of glial cells, fueling an inflammatory loop that may promote neuronal injury and memory decline (8).

## Intravascular NETs in AD

The release of intravascular NETs by adherent neutrophils has been observed in several diseases, including sepsis, atherosclerosis, autoimmune pathologies, such as autoimmune small-vessel vasculitis, experimental deep vein thrombosis, transfusion-related acute lung injury, and cancer (1, 4, 56–59). Intravascular NETosis can be triggered by different stimuli including microbes, pro-inflammatory cytokines, activated platelets, and antibody–antigen complexes (1, 2, 56, 60, 61).

**Figure 1:**
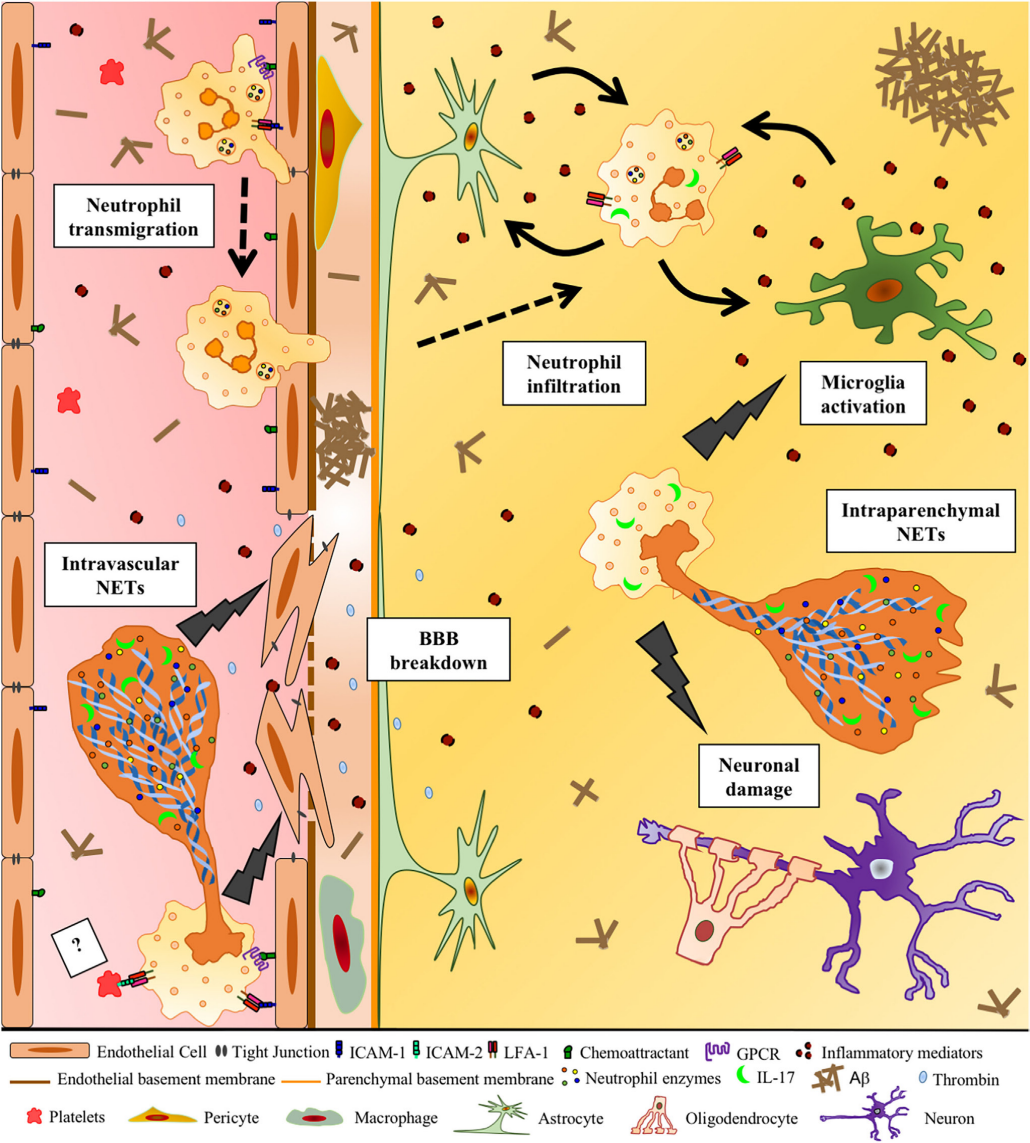
**Potential deleterious effects of neutrophil extracellular traps (NETs) in Alzheimer’s disease (AD)**. Aβ and other DAMPs and pro-inflammatory factors lead to the activation of cerebral endothelial cells, inducing the upregulation of adhesion molecules and chemoattractants on these cells. This allows circulating neutrophils to adhere intravascularly *via* LFA-1 β2 integrin–ICAM-1 binding and then extravasate into the brain. Chemoattractants on the surface of the endothelium may activate β2 integrins *via* inside–out signaling triggered by G-protein-coupled seven-pass transmembrane receptors (GPCRs), allowing neutrophil arrest. Neutrophils that adhere to the vessel wall produce intravascular NETs, potentially with the contribution of activated platelets, probably through the binding of neutrophil LFA-1 with platelet ICAM-2 (?). DNA, histones, IL-17, active proteases, such as MPO, neutrophil elastase, and cathepsin G, released during NET formation, induce the production of pro-inflammatory cytokines and thrombin, contributing to the loss of blood–brain barrier integrity. Neutrophils inside the cerebral parenchyma are activated by inflammatory mediators potentially released by glial cells and produce NETs, which may further activate glial cells and harm surrounding neurons. NETs could, therefore, represent a mechanism of neutrophil-mediated intravascular and intraparenchymal tissue damage in AD.

The intravascular neutrophil adhesion cascade that causes neutrophils to leave the blood circulation begins with the capture of these cells on the endothelium followed by their rolling and firm arrest on activated endothelial cells (1, 25). Adhesion receptors specialized for the arrest of intravascular neutrophils are heterodimeric transmembrane proteins known as integrins. In order to mediate firm adhesion, integrins undergo an activation process induced by chemoattractants *via* G-protein-coupled receptors. The activation of the intracellular pathways leading to increased ligand binding affinity (“integrin activation”) and the clustering of integrins in the membrane, which together allow cell attachment, is defined as “inside–out signaling” (62). Furthermore, the signaling steps that occur following the ligand-induced clustering of leukocyte integrins are described as the “outside–in pathway” (62). Indeed, β2 integrin engagement stabilizes neutrophil adhesion to the inflamed endothelium and induces neutrophils to release ROS, cytotoxic enzymes, arachidonic acid derivatives, cytokines, and chemoattractants, which may have a detrimental effect on the vessel wall (53, 55). Previous studies including our own have also shown that neutrophil adhesion on the vessel wall without transmigration is sufficient to induce endothelial injury, suggesting that intravascular adhesion *per se* may trigger NET formation and consequent endothelial injury, compromising BBB integrity (54, 55, 63). The adhesion-dependent production of ROS may also trigger the machinery involved in the final extrusion of fibers containing DNA and granule proteins (5). The adhesion of neutrophils to the endothelium induced by the engagement of αMβ2 integrin (Mac-1) promotes the release of NETs by neutrophils in the presence of lipopolysaccharide (64). In addition, the activation of αMβ2 integrin induces changes in the neutrophil cytoskeleton that facilitate the breakdown of nuclear and plasma membranes, favoring the release of NETs (64). The engagement of LFA-1, another β2 integrin (αLβ2 heterodimer) expressed by neutrophils, triggers NET formation by activated platelets *in vitro* and *in vivo* (Figure 1) (59). During inflammation, Mac-1 and LFA-1 mediate interactions with vascular ICAM-1, and microvascular endothelial cells produce higher levels of ICAM-1 in both transgenic AD mouse models and human AD patients (8, 25). Interestingly, we have recently reported the expression of adhesion molecules in areas burdened by Aβ plaques and rich in migrated leukocytes in animal models of AD (8). Accordingly, *in vitro* studies have demonstrated that Aβ peptides induce the expression of endothelial adhesion molecules, including ICAM-1 in mouse and human brain endothelial cells, suggesting that Aβ may play a role in endothelial activation and intravascular neutrophil adhesion in AD (8, 65). Our recent data indicate that both oligomeric and fibrillary Aβ1–42 trigger the rapid, integrin-dependent adhesion of human and mouse neutrophils on fibrinogen and ICAM-1, a ligand for LFA-1 integrin (8). Moreover, we have shown that Aβ1–42 induces both the intermediate-affinity and high-affinity states of LFA-1, potentially providing stop signals for neutrophils. In addition, our recent data indicate that neutrophils migrate into the brains of AD mouse models by engaging LFA-1 integrin and that blocking this β2 integrin prevents neutrophil adhesion in cortical venules and subsequent extravasation, suggesting intravascular neutrophil adhesion through β2 integrins may trigger the formation of NETs in AD (8). We also have recently confirmed the formation of intravascular NETs in 5xFAD and 3xTg-AD mice and proposed as a mechanism for neutrophil-dependent damage in AD (Figure 1) (8). Furthermore, we have documented intravascular neutrophil adhesion and the release of NETs in the brains of human AD subjects, suggesting that intravascular NETs may also contribute to CNS damage in humans.

Previous studies have shown that pro-inflammatory cytokines, such TNFα, IL-1β, and IL-8, can be released by activated endothelial cells and may, therefore, trigger intravascular NETs (60, 66–68). The cerebral vasculature in human AD subjects is strongly activated and produces cytokines, suggesting that intravascular cytokines may favor the formation of NETs in this context. AD brain microvessels release significantly higher levels of thrombin, TNFα, IL-1β, and IL-8 than age-matched controls, indicating that such endothelial molecules may promote the formation of NETs by adherent neutrophils (69–71). Thrombin in particular becomes more abundant in the cerebral capillaries of AD brains, and induces the release of IL-1β and IL-8, which may in turn contribute to intravascular NETosis (60, 70–73). Interestingly, subjects with mild cognitive impairment have higher serum IL-1β levels than controls, suggesting this cytokine may trigger the release of NETs and contribute to the onset of AD (74). Previous studies have also shown elevated levels of IL-1β and TNFα in the serum of AD patients (74–76). *In vitro* studies of brain endothelial cells indicate that exposure to Aβ peptides increases the expression of cytokine genes, including the gene encoding IL-1β, which induces NETosis (77). Aβ may further contribute to intravascular NETosis through its interaction with the receptor for advanced glycation end-products (RAGE) on brain endothelial cells, promoting the generation of ROS and the secretion of pro-inflammatory cytokines (19, 65, 78).

Several studies have shown that platelets exist in a pre-activated state in the blood of an AD mouse model (APP23) and in human AD patients, showing strongly enhanced responses upon stimulation and potentially offering biomarkers for the early diagnosis of AD (79, 80). The exposure of platelets to Aβ induces platelet activation with the further production of Aβ and ROS, initiating a vicious circle that enhances vascular inflammation (81, 82). Activated platelets can also trigger the production of NETs, and *in vitro* studies have demonstrated that adding platelets stimulated with agonists, such as ADP, collagen, thrombin, LTB4, or arachidonic acid to neutrophils causes NET formation (58, 83, 84). Intravascular NETs may be induced by activated platelets interacting with neutrophils *via* Toll-like receptor 4 (TLR4) or LFA-1 integrin, and we speculate that the release of intravascular NETs found in AD mouse models and human AD subjects could be promoted by activated platelets interacting with adherent neutrophils (56, 59, 85). Interestingly, blocking LFA-1 integrin inhibits neutrophil adhesion in the brain microvasculature of AD mice, and LFA-1 deficiency reduces the cognitive deficit and neuropathological changes in animal models of AD (8). However, it is unclear whether intravascular NETosis is less severe in AD mice lacking LFA-1 integrin, and further studies are required to address this issue. Following activation, platelets present the high mobility group box 1 (HMGB1) protein to neutrophils, causing them to produce NETs (83). HMGB1 is a damage-associated molecular pattern (DAMP) released during apoptosis and is involved in leukocyte recruitment and local activation (86). It migrates from the cytoplasm to the surface following platelet activation and interacts with several receptors on the neutrophil surface including RAGE (87). RAGE has been shown to play an essential role in the production of NETs and the treatment of neutrophils with anti-RAGE antibodies prevents NET formation induced either by activated platelets or by recombinant HMGB1, suggesting that Aβ or HMGB1 inside vessels may interact with neutrophil RAGE leading to NET formation in AD (83). HMGB1 activates neutrophils, induces the production of pro-inflammatory cytokines and upregulates the expression of VCAM-1 and selectins on endothelial cells, potentially amplifying the inflammatory responses in AD (87–90). HMGB1 also potentiates further NET formation by interacting with TLR4 in a ROS-independent manner, contributing to tissue damage during sterile inflammation (91). We, therefore, hypothesize that the release of HMGB1 during the formation of NETs may exacerbate neuroinflammation and that HMGB1-targeted therapy may, therefore, be beneficial in neutrophil-associated inflammatory conditions, such that blocking the activity of this protein may also offer a new therapeutic approach to AD.

Intravascular NETosis promotes blood clotting, and NET release by activated neutrophils triggers both thrombin formation through the induction of prothrombinase activity and the aggregation of platelets (57, 92, 93). Indeed, during severe sepsis in liver sinusoids, intravascular NETs induce thrombus formation and the partial or total occlusion of capillaries (59). Thrombin expressed by endothelial cells enhances platelet activation, amplifying chronic inflammation and thrombus formation (94). Thrombin levels are elevated in the vessel walls and senile plaques of AD patients, and thrombin inhibitors can reduce vascular inflammation by limiting the cerebrovascular expression of inflammatory proteins and ameliorating cognitive functions in transgenic animal models of AD (71, 95). Thrombin formation triggered by NETosis may, therefore, exacerbate vascular inflammation and neuronal injury in AD. Furthermore, NETs and IL-17 are important constituents of the fresh and lytic thrombus after acute myocardial infarction, and their specific co-localization suggests that they may play a role during thrombus stabilization and growth (96). Platelets express the receptor for IL-17A and IL17-F (IL–17RA), and the incubation of platelets with IL-17A promotes their aggregation (97). Notably, elevated IL-17 serum levels have been reported in a cohort of Chinese AD patients, suggesting it may contribute to platelet activation during AD (98). Altogether, these findings suggest that the formation of NETs together with IL-17 release by neutrophils may activate platelets and exacerbate brain microvessel pathology, contributing to the reduced brain perfusion and NVU alterations observed in AD (99).

Intravascular NETs can also damage the endothelial wall by releasing a mixture of nuclear proteins and proteases, NE, cathepsin G, and metalloproteinases (MMPs). Indeed, NE and MMPs may destroy tight junction components to promote endothelial cell injury (100). As recently reported in patients with SLE, the MMPs normally contained within neutrophil granules are externalized in NETs and can damage the integrity of the vascular wall, and MMP-9 in particular can activate endothelial MMP-2 and trigger apoptosis (101). In addition, NE increases endothelial permeability and the expression of ICAM-1 on endothelial cells and thus can damage the BBB (102). The most abundant proteins in NETs are MPO and histones, and these can also induce endothelial cell death (103, 104). These vasculopathic effects of NETs have been demonstrated *in vivo* by using protein arginine deiminase (PAD) inhibitors to prevent the formation of NETs, which protects against vascular damage and ameliorates the phenotype of SLE (105, 106). In AD mouse models, the expression of MMPs is induced whereas the expression of tight junction proteins is suppressed in microvessels near Aβ plaques in the brain (107). Furthermore, the treatment of the BBB with oligomeric Aβ1–42 *in vitro* increased its permeability and reduced the availability of tight junction scaffold proteins (108). MMP-2 and MMP-9 released by endothelial cells stimulated with Aβ1–42 contribute to Aβ-induced BBB leakage, so the MMPs externalized during NET formation may exacerbate tight junction damage and changes in BBB permeability induced by Aβ (108).

## Intraparenchymal NETosis in AD

Neutrophils invade the brain parenchyma of AD mouse models at the early stage of AD and contribute to the induction of memory deficit (8, 32). We have recently shown that intraparenchymal migrating neutrophils produce NETs, showing the presence of cells releasing MPO, NE, and citrullinated histone H3 in the parenchyma of mouse models of AD (8). Furthermore, we have also confirmed the formation of NETs in human AD subjects by the co-localization of MPO and citrullinated histones, and of MPO and NE (8). These data suggest that NETs may represent a neutrophil-dependent disease mechanism in patients with AD (Figure 1).

Our recent TPLSM data indicate that a significant proportion of the intraparenchymal neutrophils are fully arrested, suggesting the presence of activating stop signals for neutrophils inside the brains of mouse AD models. Neutrophils migrate inside the parenchyma in areas with Aβ plaques and less neuronal fluorescence, suggesting a role for Aβ in neutrophil migration inside the parenchyma and in providing stop signals for neutrophils. Aβ is included in the class of DAMP that are released following non-microbial tissue injury, alerting the innate immune system and activating a wide array of receptors and pro-inflammatory pathways (109).

Aβ promotes the generation of ROS by activating NADPH oxidase in both human and mouse neutrophils *in vitro*, and several reports, including our own, have demonstrated that ROS production is a necessary step in the formation of NETs (5, 8, 110). These data provide further support for the role of Aβ in intraparenchymal NET formation and neutrophil-dependent CNS damage during AD. Interestingly, a recent study demonstrated that NET formation in human neutrophils *in vitro* is also driven by the fibrillary form of amyloids from other sources, such as α-synuclein, Sup35, and transthyretin (111). In the same study, the presence of NETs was observed near amyloid deposits in patients with systemic amyloidosis, and NET-associated elastase was able to degrade amyloid fibrils into short toxic oligomeric species, suggesting that amyloid fibrils act as a reservoir of toxic peptides that may promote amyloid disease pathogenesis. We, therefore, hypothesize that Aβ may trigger NET formation in the AD brain by binding to FPR1 or FPR-like-1 receptors on neutrophils, and the NETs may in turn promote the release of toxic Aβ species from amyloid plaques, amplifying the inflammatory network in AD.

Intraparenchymal cytokines, such as TNFα, IL-1β, and IL-8, produced by neural cells may also promote NET formation in extravasated neutrophils during AD (60). Indeed, our recent data suggest that interconnectivity between neutrophils and glial cells may create several feedback loops, amplifying and sustaining their reciprocal activation (Figure 1). Accordingly, activated astrocytes and microglia in AD patients secrete pro-inflammatory cytokines, such IL-1β, TNFα, and IL-8, as well as ROS into the surrounding brain tissue rich in Aβ deposits, thus potentially contributing to intraparenchymal NET formation and generating crosstalk with intraparenchymal neutrophils (Figure 1) (12, 13, 17). Both TNFα and IL-1β have recently been implicated in NETosis in rheumatoid arthritis and gout, suggesting they may contribute to NET formation also in AD (112, 113). Moreover, treatment with anakinra (a recombinant IL-1 receptor antagonist) or a monoclonal antibody that blocks IL-1β caused the partial inhibition of NET formation by neutrophils treated with synovial fluid from patients with gout, further supporting a role for IL-1β in the formation of NETs (112). Higher levels of IL-1β and TNFα are found in the brain and cerebrospinal fluid (CSF) of AD patients, suggesting these molecules may contribute to neutrophil activation and NET formation in AD (114, 115). IL-8 is abundant in the CSF of patients in the prodromal stage of AD and in the brains of AD subjects, suggesting this cytokine may attract neutrophils and contribute to NET formation in the AD brain (116). Our recent data showed that migrating neutrophils produce IL-17 in the cortex and hippocampus of 3xTg-AD mice (8). IL-17 is a cytotoxic cytokine for neurons and may contribute to the loss of BBB integrity and the recruitment of neutrophils in other inflammatory CNS diseases (117, 118). Furthermore, recent studies show that IL-17 contributes to NETosis in rheumatoid arthritis and in a model of acute myocardial infarction, suggesting this cytokine may favor NET formation also in AD (96, 113). These combined data suggest that pro-inflammatory cytokines may act in concert with Aβ and ROS to promote intraparenchymal NETosis in AD.

Intraparenchymal NETosis may be harmful to neural cells in AD through several mechanisms (Figure 1). Indeed, during the generation of NETs, the azurophilic granules of neutrophils release MMPs, in particular MMP-9, and serine proteases such as NE, cathepsin G, and MPO, which can induce tissue damage and aggravate the inflammatory process. Neutrophils are equipped with high levels of MMP-9, stored as the inactive form pro-MMP-9. Recent data indicate that that Aβ25–35 induces the degranulation process following neutrophil activation and the massive secretion of the inactive pro-MMP-9 stored in cytoplasmic granules (119). After neutrophil stimulation, pro-MMP-9 can be converted into active MMP-9 by several of the proteases released by activated neutrophils (including NE), or by Aβ-stimulated brain cells (119). MMPs are involved in the proteolysis of the extracellular matrix and can thus damage the brain parenchyma (120). NE can also induce the degradation of tissues not only by cleaving extracellular matrix proteins, such as elastin, collagen, and proteoglycan but also by activating MMPs and inactivating the endogenous tissue inhibitors of MMPs (TIMPs) (121). MPO localized within NETs also inactivates TIMPs and thus indirectly enhances the local pathogenic activity of MMPs (122). TIMPs have been localized in neuritic senile plaques and NFTs in the hippocampus and cerebral cortex of human AD brains (123). Furthermore, MMP-9 is expressed in senile plaques, NFTs, and the vascular walls of human AD brains as well as in Aβ-stimulated astrocytes and activated microglia, and its inhibition is therapeutically beneficial in a transgenic mouse model of AD (124–127). The main constituents of NETs are histones (2). Each histone protein has an N-terminal tail with lysine and arginine residues that extend from the core. These residues can be modified by acetylation, methylation, and citrullination among others, and the latter is associated with PAD4, which plays a central role in the formation of NETs. In PAD4-knockout mice, the absence of histone citrullination prevents the decondensation of chromatin (64, 128). When translocated into the extracellular space, histones function as DAMP, amplifying the sterile inflammation state and showing toxicity to the surrounding cells by activating TLRs and inflammasome pathways (129, 130). During neurodegeneration in particular, extracellular histones can stimulate the innate immune response and induce apoptosis in neuronal cells. Indeed, a recent study has shown that extracellular histone H1 induces a pro-inflammatory response in microglia and causes neuronal death by activating the mitochondrial apoptosis pathway (131). In AD brains, extracellular histone H1 has been found within amyloid plaques due to its capacity to bind APP and β-amyloid with high affinity (132, 133). The accumulation of extracellular histones may, therefore, accelerate neurodegeneration and perpetuate the inflammatory process in AD.

## Future Directions

NETosis aggravates several inflammatory and autoimmune disorders. The unexpected recent discovery that neutrophils promote AD pathogenesis in mouse models opened a new area of investigation highlighting the prominent role of circulating immune system cells in AD. The mechanisms of neutrophil-dependent damage in AD are unclear, and the discovery of NETs in mouse models of AD and human AD patients indicates a potential mechanism that neutrophils may use to induce and exacerbate neuroinflammation, by promoting cerebral vasculature dysfunction and parenchymal damage. However, the role of NET components in the induction and perpetuation of neuroinflammation in AD needs to be determined in more detail in further studies.

Neutrophils are key regulators of the immune system because these cells communicate and interact with adaptive immune system cells during infections and chronic inflammatory and autoimmune diseases (33, 134). NETs activate plasmacytoid dendritic cells through TLR9 during viral infections and autoimmune diseases and can mediate the priming of T cells, which requires NET–T cell contacts and T-cell receptor signaling (7, 134, 135). Adaptive immune system cells are encountered in the AD brain, and they may play a role in AD pathogenesis, but it is unclear whether NETs link the innate and adaptive immune responses in AD (21). NET components provide a source of autoantigens, which promote the production of autoantibodies and stimulate the immune system leading to tissue damage, but it is not known whether NETs are autoantibody targets and whether they generate AD biomarkers (4, 113). Citrullinated proteins in particular bind to autoantibodies, which in association with pro-inflammatory cytokines perpetuate the formation of NETs (113, 136). The presence of citrullinated vimentin and histone H3 has been detected in the hippocampus and cerebral vessels of AD patients, but the role of such proteins in AD is currently unclear (8, 137).

The inhibition of NET formation could offer a novel therapeutic approach to limit the extensive damage caused during AD. Evidence from animal models and human AD patients suggests that the targeting of NET components, such as NADPH oxidase, PAD, and DNase I, may help to prevent NET extrusion and limit tissue damage (138). Indeed, NET-targeted therapy has shown beneficial effects in animal models of diseases, such as SLE, atherosclerosis, and rheumatoid arthritis (105, 113, 138, 139). However, the effect of blocking NET formation in animal models of AD has not yet been demonstrated and further studies are required to determine whether this approach has merit. In conclusion, NETs represent a novel disease mechanism in AD, and targeting their effects during sterile inflammation may provide an additional therapeutic strategy for the treatment of this devastating disease.

## Author Contributions

The authors equally contributed to this work.

## Conflict of Interest Statement

The authors declare that the research was conducted in the absence of any commercial or financial relationships that could be construed as a potential conflict of interest.
